# Supplementary material for: Deep Evolutionary History of the Phox and Bem1 (PB1) Domain Across Eukaryotes
Source: Sci Rep. 2020 Mar 2;10:3797. doi: 10.1038/s41598-020-60733-9 (PMC7051960; doi:10.1038/s41598-020-60733-9)
Supplement: Supplementary file 1 — Supplementary Information. [file 41598_2020_60733_MOESM1_ESM.pdf]

Supplementary material to:

Deep Evolutionary History of the Phox and Bem1 (PB1) Domain Across Eukaryotes

Sumanth Kumar Mutte[1] and Dolf Weijers[1, *]

This document contains:

- 4 Supplementary Figures
- 3 Supplementary Tables

Additional Files 1-5 are available online.

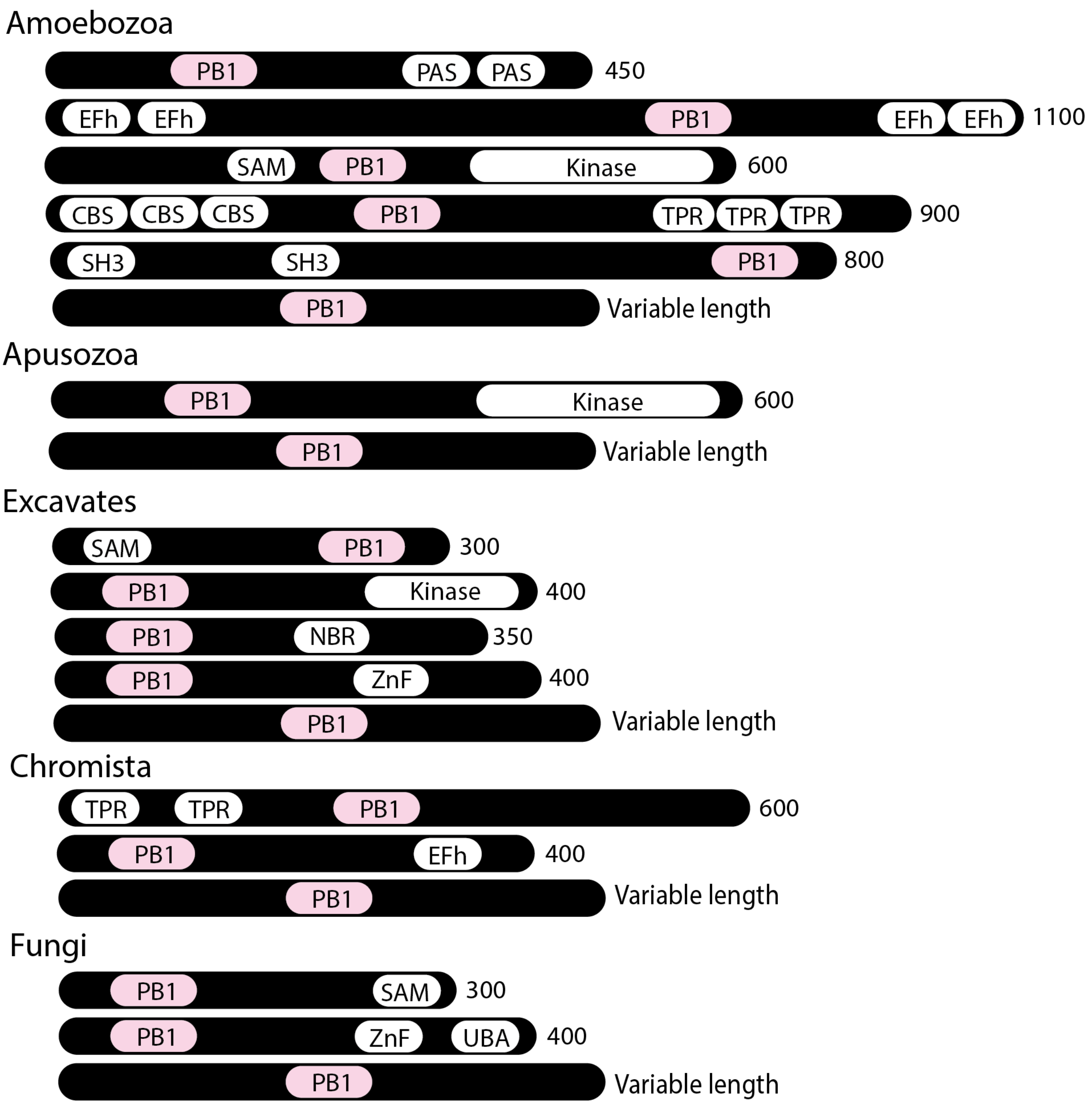

Fig. S1: Presence of various PB1 domain containing proteins identified in only one sequence and/or one species. The numbers at the end of each row is the approximate length of the protein. The complete information about the domains and their respective InterPro database links are provided in Table S1.

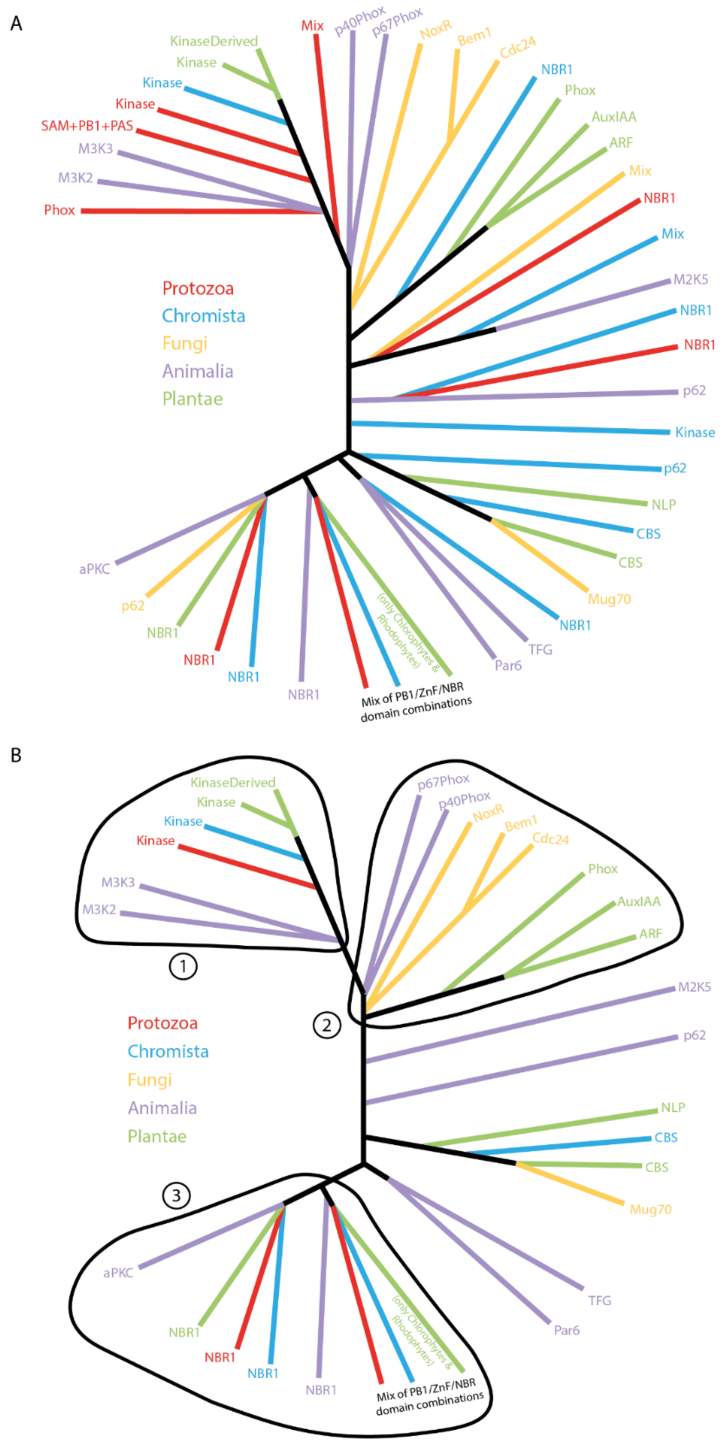

Fig. S2: Complete (A) and simplified (B) illustration of the unrooted tree with the PB1 domains from all five kingdoms. Orthologs from each kingdom are represented with each colour as indicated: Protozoa in 'red', Chromista in 'blue', Fungi in 'orange', Animalia in 'purple' and Plantae in 'green'. The groups outlined with continuous lines indicated with numbers 1, 2 and 3 represent the probable ancestral copies in LECA corresponding to Kinase, Phox and NBR1 groups respectively. 'Mix' indicates a combination of (partial) PB1 domains with other domains in random. Full version of the tree with taxa names and domain information can be found at iTOL: https://itol.embl.de/shared/dolfweijers.

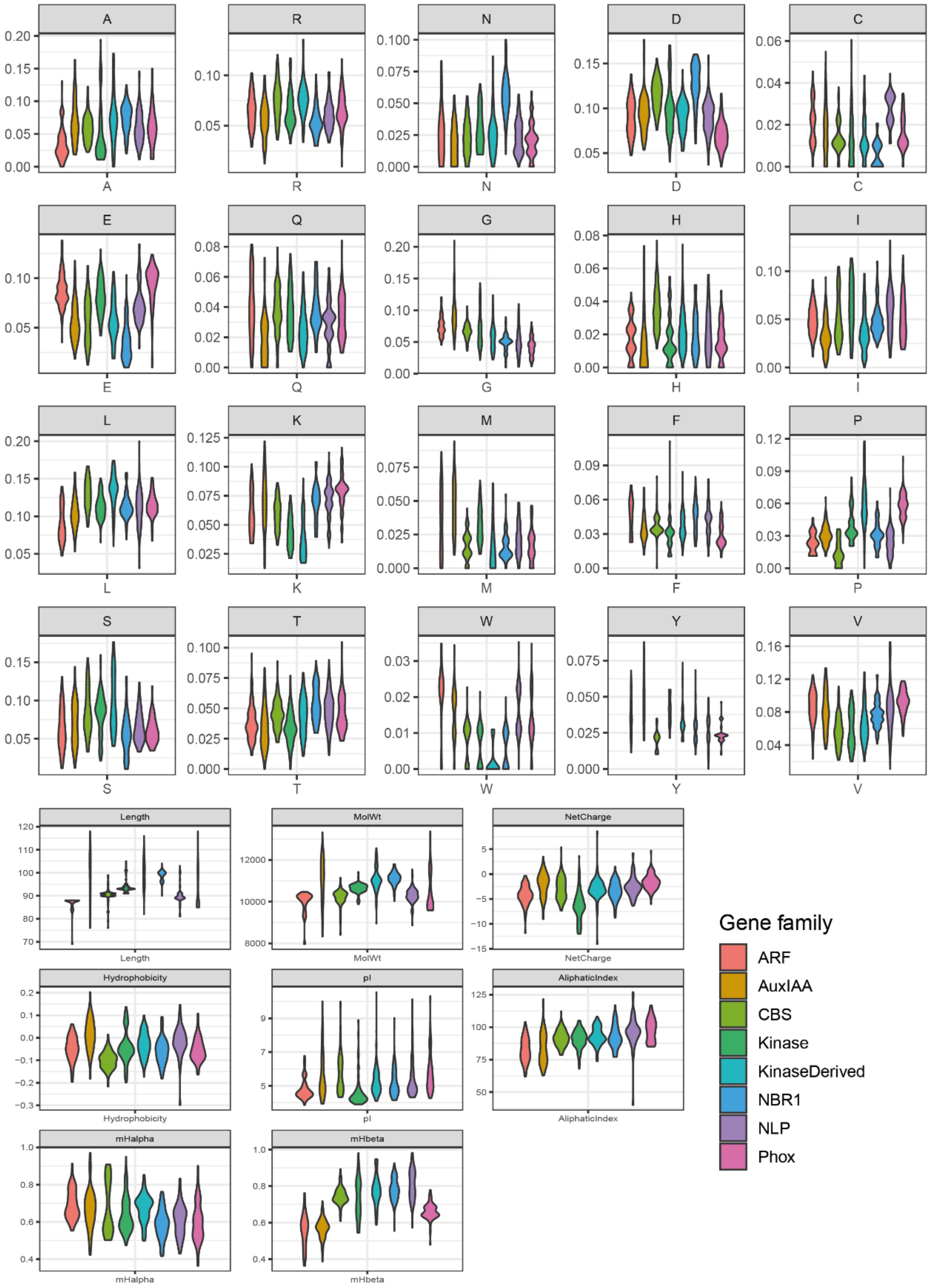

Fig. S3: Violin plots showing the descriptive stats of the 28 descriptors/variables used Random Forest (RF) classification. Colours in each plot represent one gene family as shown in the legend.

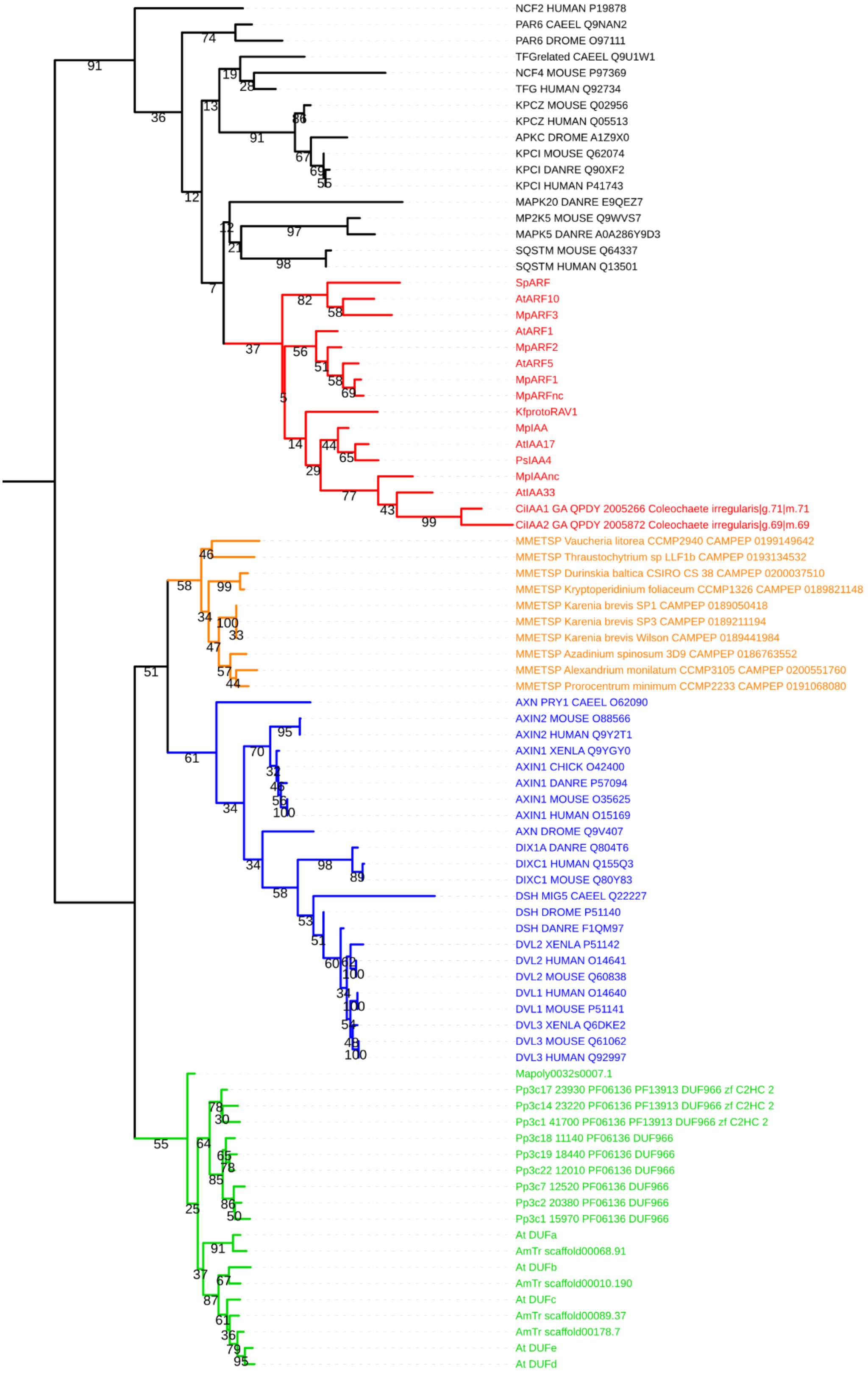

Figure S4: DIX and PB1 domain based phylogenetic tree. Branch/label colors represent the two phylogenetic groups they belong to: (1) Animal PB1(black); Plant ARF/AuxIAA PB1 (red); and (2) SAR group DIX (orange); Animal DIX (blue); Plant DIX (green).

SUPPLEMENTARY TABLES

Table S1: List of domains (present in Fig. 1) with their short name, full name and a link to the InterPro domain database.

ShortName	FullName (InterProID)	InterProDB link
PB1	PB1 domain (IPR000270)	https://www.ebi.ac.uk/interpro/entry/IPR000270
Kinase	Protein kinase domain (IPR000719)	https://www.ebi.ac.uk/interpro/entry/IPR000719
WD40	WD40 repeat (IPR001680)	https://www.ebi.ac.uk/interpro/entry/IPR001680
TPR	Tetratricopeptide repeat (IPR019734)	https://www.ebi.ac.uk/interpro/entry/IPR019734
ZnF	Zinc finger, ZZ-type (IPR000433)	http://www.ebi.ac.uk/interpro/entry/IPR000433
CentralDomain	Next to BRCA1, central domain (IPR032350)	http://www.ebi.ac.uk/interpro/entry/IPR032350
UBA	Ubiquitin-associated domain (IPR015940)	http://www.ebi.ac.uk/interpro/entry/IPR015940
CBS	CBS domain (IPR000644)	http://www.ebi.ac.uk/interpro/entry/IPR000644
RWPRK	RWP-RK domain (IPR003035)	http://www.ebi.ac.uk/interpro/entry/IPR003035
DH	Dbl homology (DH) domain (IPR000219)	http://www.ebi.ac.uk/interpro/entry/IPR000219
PH	Pleckstrin homology domain (IPR001849)	http://www.ebi.ac.uk/interpro/entry/IPR001849
SH3	SH3 domain (IPR001452)	http://www.ebi.ac.uk/interpro/entry/IPR001452
PX	Phox homologous domain (IPR001683)	http://www.ebi.ac.uk/interpro/entry/IPR001683
kDAG	Phorbol ester/diacylglycerol-binding domain (IPR002219)	http://www.ebi.ac.uk/interpro/entry/IPR002219
PDZ	PDZ domain (IPR001478)	http://www.ebi.ac.uk/interpro/entry/IPR001478
ARF-DBD/B3	B3 DNA binding domain (IPR003340)	http://www.ebi.ac.uk/interpro/entry/IPR003340
ARF-DBD/ARF	Auxin response factor (IPR010525)	http://www.ebi.ac.uk/interpro/entry/IPR010525
Aux/IAA-I	Domain-I or EAR motif	NA
Aux/IAA-II	Domain-II or DEGRON motif	NA

Table S2: List of identifiers of the PB1 domain containing proteins from four species of land plants (Marchantia, Physcomitrella, Amborella and Arabidopsis) used for the sequence alignment and logo construction.

GeneFamily	Marchantia	Physcomitrella	Amborella	Arabidopsis
ARF	Mapoly0019s0045.1	Pp3c1_14480V3.1	evm_27.model.AmTr_v1.0_scaffold00007.382	AT1G59750
	Mapoly0011s0167.1	Pp3c1_40270V3.1	evm_27.model.AmTr_v1.0_scaffold00016.128	AT5G62000
	Mapoly0075s0050.1	Pp3c2_25890V3.1	evm_27.model.AmTr_v1.0_scaffold00021.210	AT5G60450
		Pp3c4_12970V3.3	evm_27.model.AmTr_v1.0_scaffold00025.251	AT1G19850
		Pp3c4_13010V3.3	evm_27.model.AmTr_v1.0_scaffold00029.187	AT1G30330
		Pp3c5_9420V3.1	evm_27.model.AmTr_v1.0_scaffold00057.126	AT5G20730
		Pp3c6_21370V3.1	evm_27.model.AmTr_v1.0_scaffold00092.36	AT5G37020
		Pp3c13_4720V3.1	evm_27.model.AmTr_v1.0_scaffold00148.24	AT4G23980
		Pp3c14_16990V3.10	evm_27.model.AmTr_v1.0_scaffold00155.56	AT2G28350
		Pp3c16_6100V3.1	evm_27.model.AmTr_v1.0_scaffold00211.4	AT2G46530
		Pp3c17_19900V3.1		AT1G34310
		Pp3c27_60V3.1		AT1G34170
		Pp3c9_21330V3.1		AT1G35540
		Pp3c15_9710V3.1		AT1G35520
				AT4G30080
				AT3G61830
				AT1G19220
				AT1G35240
				AT1G34410
				AT1G34390
AuxIAA	Mapoly0013s0010.1	Pp3c24_6610V3.1	evm_27.model.AmTr_v1.0_scaffold00002.512	AT4G14560
	Mapoly0034s0017.1	Pp3c8_14720V3.1	evm_27.model.AmTr_v1.0_scaffold00002.514	AT3G23030
			evm_27.model.AmTr_v1.0_scaffold00019.282	AT1G04240
			evm_27.model.AmTr_v1.0_scaffold00039.160	AT5G43700
			evm_27.model.AmTr_v1.0_scaffold00045.141	AT1G15580
			evm_27.model.AmTr_v1.0_scaffold00056.118	AT1G52830
			evm_27.model.AmTr_v1.0_scaffold00109.120	AT3G23050
			evm_27.model.AmTr_v1.0_scaffold00122.5	AT2G22670
			evm_27.model.AmTr_v1.0_scaffold00184.12	AT5G65670
				AT1G04100
				AT4G28640
				AT1G04550
				AT2G33310
				AT4G14550
				AT3G04730
				AT1G04250
				AT1G51950
				AT3G15540
				AT2G46990
				AT3G16500
				AT4G29080
				AT5G25890
				AT4G32280
				AT3G62100
				AT3G17600

				AT2G01200
				AT5G57420
				AT1G15050
CBS	Mapoly0179s0023.1	Pp3c1_14290V3.1	evm_27.model.AmTr_v1.0_scaffold00013.41	AT5G63490
		Pp3c1_14310V3.1	evm_27.model.AmTr_v1.0_scaffold00017.72	AT2G36500
		Pp3c11_15160V3.1		AT3G52950
		Pp3c2_26110V3.1		AT5G50640
		Pp3c7_10070V3.1		
Kinase	Mapoly0013s0150.1	Pp3c15_24250V3.1	evm_27.model.AmTr_v1.0_scaffold00004.293	AT1G04700
		Pp3c9_25280V3.1	evm_27.model.AmTr_v1.0_scaffold00019.236	AT1G16270
			evm_27.model.AmTr_v1.0_scaffold00026.90	AT1G79570
			evm_27.model.AmTr_v1.0_scaffold00039.196	AT2G35050
			evm_27.model.AmTr_v1.0_scaffold00081.26	AT3G24715
				AT3G46920
				AT5G57610
KinaseDerived			evm_27.model.AmTr_v1.0_scaffold00007.258	AT1G25300
			evm_27.model.AmTr_v1.0_scaffold00046.178	AT1G70640
			evm_27.model.AmTr_v1.0_scaffold00049.221	AT2G01190
			evm_27.model.AmTr_v1.0_scaffold00109.93	AT3G18230
				AT3G26510
				AT3G48240
				AT4G05150
				AT5G09620
				AT5G16220
				AT5G49920
				AT5G63130
				AT5G64430
NBR1	Mapoly0100s0042.1	Pp3c11_16970V3.1	evm_27.model.AmTr_v1.0_scaffold00049.238	AT4G24690
		Pp3c7_8990V3.1		
NLP	Mapoly0083s0040.1	Pp3c12_2070V3.1	evm_27.model.AmTr_v1.0_scaffold00058.115	AT2G17150
		Pp3c15_9180V3.1	evm_27.model.AmTr_v1.0_scaffold00066.150	AT4G35270
		Pp3c17_4370V3.1	evm_27.model.AmTr_v1.0_scaffold00080.66	AT4G38340
		Pp3c17_4375V3.1		AT1G20640
		Pp3c19_2670V3.1		AT1G76350
		Pp3c19_2720V3.1		AT1G64530
		Pp3c22_6360V3.1		AT4G24020
		Pp3c22_6370V3.1		AT2G43500
		Pp3c9_14600V3.1		AT3G59580

Table S3: Confusion matrix from the Random Forest (RF) model. Diagonal values represent the correctly classified PB1's, and others represent the mis-classified category. The column 'class.error' represents the classification error for that particular class of PB1's as shown in Fig. 5a.

	ARF	AuxIAA	CBS	Kinase	KinaseDerived	NBR1	NLP	Phox	class.error
**ARF**	99	0	1	0	0	0	0	0	0.01
**AuxIAA**	4	95	1	0	0	0	0	0	0.05
**CBS**	2	1	92	1	0	2	2	0	0.08
**Kinase**	0	1	0	95	4	0	0	0	0.05
**KinaseDerived**	1	0	2	7	87	0	1	2	0.13
**NBR1**	0	2	2	1	1	72	0	0	0.07
**NLP**	0	0	3	0	1	0	95	1	0.05
**Phox**	0	2	0	0	0	0	2	96	0.04
