# Supplementary figures and images for: Deep Evolutionary History of the Phox and Bem1 (PB1) Domain Across Eukaryotes
Source: Sci Rep. 2020 Mar 2;10:3797. doi: 10.1038/s41598-020-60733-9 (PMC7051960; doi:10.1038/s41598-020-60733-9)

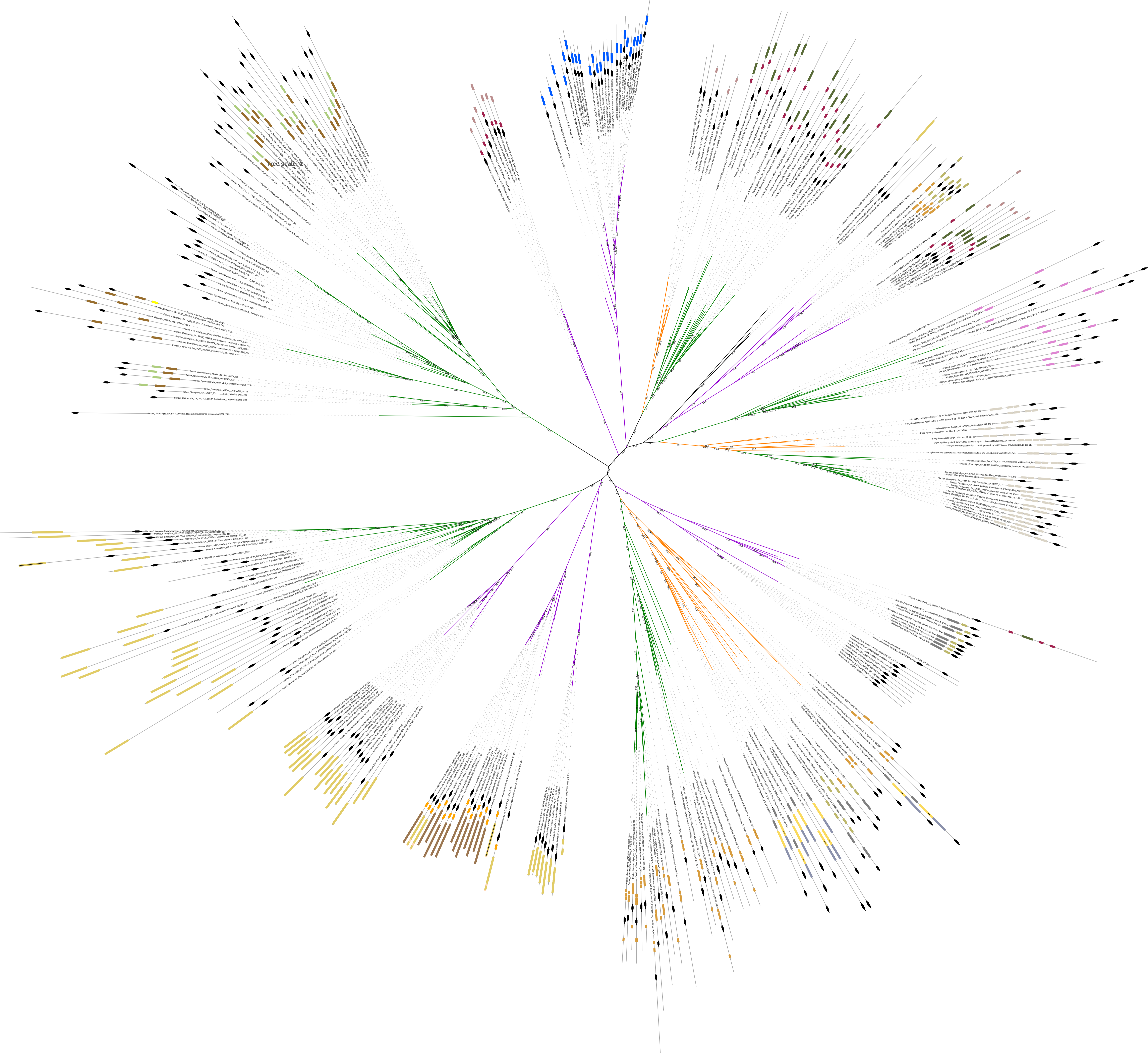

Supplement: Supplementary file 2 — Supplementary Information 2. [file 41598_2020_60733_MOESM2_ESM.pdf]

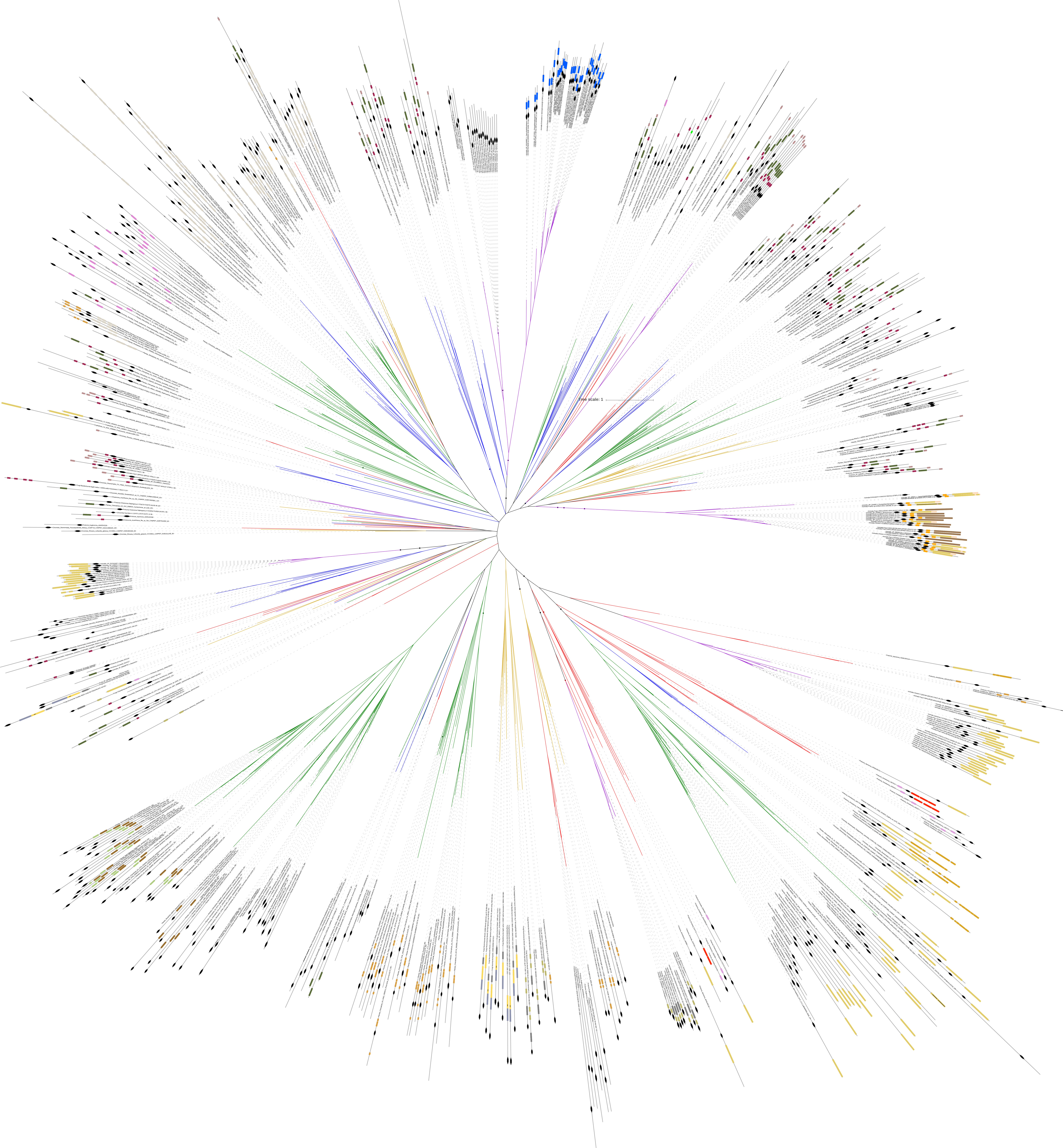

Supplement: Supplementary file 3 — Supplementary Information 3. [file 41598_2020_60733_MOESM3_ESM.pdf]
